# Effect of mild gestational diabetes mellitus on histological, ultrastructural, and quantitative morphometric alterations of rat fetal liver

**DOI:** 10.22038/ijbms.2024.78920.17069

**Published:** 2024

**Authors:** Sahar Ardalan Khales, Asghar Mafinezhad, Masoud Golalipour, Gholamreza Roshandel, Soraya Ghafari, Abdolhalim Rajabi, Mohammad Jafar Golalipour

**Affiliations:** 1 Golestan Research Center of Gastroenterology and Hepatology, Golestan University of Medical Sciences, Gorgan, Iran; 2 Gorgan Congenital Malformations Research Center, Golestan University of Medical Sciences, Gorgan, Iran; 3 Pathology Department of Shahid Kamyab Hospital, Mashhad University of Medical Sciences, Mashhad, Iran; 4 Medical Cellular and Molecular Research Centre, Golestan University of Medical Sciences, Gorgan, Iran; 5 Environmental Health Research Center, Department of Biostatistics and Epidemiology, Faculty of Health, Golestan University of Medical Sciences, Gorgan, Iran

**Keywords:** Fetus, Gestational diabetes mellitus, Histology, Liver, Morphometry, Rat, Ultrastructure

## Abstract

**Objective(s):**

Gestational diabetes mellitus (GDM), one of the most common metabolic disorders in pregnancy, impacts maternal and fetal health. This study was designed to assess the effects of mild GDM on the histology, ultrastructure, and morphometry of fetal liver tissue.

**Materials and Methods:**

In this experimental study, twenty pregnant rats were randomly allocated into control and streptozotocin (STZ)-induced diabetic groups. Mild hyperglycemia was induced by intraperitoneal injection of STZ (40 mg/kg/bw) on the 5th day of gestation. At day 19 of gestation, fetal livers were separated and subjected to histological, transmission electron microscopic, and quantitative morphometric examinations.

**Results:**

In the GDM group, PAS staining was positive, revealing scattered eosinophilic inclusions in some hepatocytes. Masson trichrome staining was also positive and showed some fibrous tissue as fine fibers in the portal spaces that extended to the central vein. Reticulin staining in the GDM group was focally positive in the areas of fibrosis and the portal spaces. Ultrastructural examination showed pyknotic nuclei, karyolysis, degranulation and vesiculation of the rough endoplasmic reticulum, and degeneration of mitochondria in the GDM group. The morphometric examination demonstrated that the mean area of hepatocytes was significantly lower in the GDM group than in the control group (*P*<0.05). Moreover, the mean diameter of the central vein and the density of megakaryocytes were significantly higher in the GDM group than in the control group (*P*<0.05).

**Conclusion:**

Uncontrolled mild GDM induced the histological, ultrastructural and morphometric alterations in the fetal liver.

## Introduction

Diabetes mellitus (DM), as one of the leading causes of death in the world, imposes a heavy global burden on public health (1). 

Gestational diabetes mellitus (GDM) is the most common medical complication and metabolic disturbance of pregnancy (2). It has attracted considerable research attention due to its severe risks and adverse health effects. 

Untreated GDM can lead to short and long-term complications for the mother and fetus, including gestational hypertension, cesarean section, preeclampsia, shoulder dystocia, birth trauma, macrosomia, neonatal hypoglycemia, hypocalcemia, and hyperbilirubinemia (3, 4). 

The elevated transfer of glucose from the mother with DM to the fetus leads to hyperglycemia and hyperinsulinemia in the fetus, which in turn stimulates the growth of insulin-dependent tissues and organs such as the liver (5-9).

Despite extensive studies on the adverse effects of DM, limited data are available regarding the impact of mild GDM on the fetal liver structure. In the present study, an attempt was made to investigate the effects of mild GDM on the histology, ultrastructure, and morphometry of fetal liver tissue in streptozotocin (STZ)-induced diabetic rats as an experimental model.

## Materials and Methods

This study was approved by the Ethics Committee of Golestan University of Medical Sciences, Gorgan, Iran (IR.GOUMS.AEC.1401.007). The experimental procedures were conducted in line with the Guide for the Care and Use of Laboratory Animals.

***Experimental design***

Adult male and female Wistar rats were used for this study. Rats were mated overnight (2 females: 1 male). Copulation was confirmed by the presence of sperm in the vaginal smear the following morning (gestational day 0) (10-12). Twenty pregnant rats were randomly allocated into control and STZ-induced diabetic groups.

***Induction of DM***

Mild hyperglycemia was induced in pregnant rats by intraperitoneal administration of STZ (Sigma, USA), with a dose of 40 mg/kg body weight, freshly dissolved in citrate buffer (pH 4.5), on the 5th day of gestation (13, 14). The rats in the control group received an equivalent volume of citrate buffer. 

The diabetic state was verified by measuring the fasting blood glucose level (120–300 mg/dl) (15) using an Accu-Chek Active glucometer 72 hr after STZ injection. The normal fasting blood glucose level (<120 mg/dl) was also checked in rats in the control group.

***Histological studies***

*Light microscopy*

Six pregnant rats from each group were sacrificed using ketamine (90 mg/kg body weight) and xylazine (10 mg/kg body weight) anesthesia mixture on day 19 of pregnancy. Then the fetal livers were removed and fixed in 10% neutral-buffered formalin. The specimens were then dehydrated in an ascending series of ethanol, cleared with xylene, and embedded in paraffin. Four to five micrometer-thick sections were stained with periodic acid-Schiff (PAS), Masson trichrome, and reticulin. Hematoxylin and eosin (H&E) staining was used for quantitative morphometric analysis.

Sections were observed under an OLYMPUS BX51 microscope, and images were taken with an OLYMPUS DP12 digital camera.

*Electron microscopy*

For the electron microscopic study, samples were fixed in 2.5% glutaraldehyde (PBS-based EM grade) and processed as per the standard protocol (16). Sections were examined under a transmission electron microscope (TEM) (Leo 912 AB, Germany).

***Morphometric study and statistical analysis***

H&E-stained sections at 4 μm thickness were used for quantitative morphometric analysis. Measurements were done using five non-overlapping fields from 10–20 sections.

***Parameters were examined***

*Using 100X oil-immersion objective*

*a. *Area and diameter of hepatocyte

b. Area and diameter of nucleus

c. Diameter of central vein

d. Diameter of bile duct

*Using 40X objective: Density of megakaryocytes in 100000 μm* ^2^ *of fetal liver*

Morphometric parameters were measured using Olysia Bio software (Olympus Optical Co. LTD, Tokyo-Japan).

***Statistics analysis***

Results were presented as mean ± standard deviation (SD). The Shapiro-Wilk test was used to evaluate the normality of the data. Comparing means of variables between groups performed by the independent two-sample t-test. The correlation between variables was determined by Pearson’s correlation coefficient test. All statistical analyses were carried out using Stata Version 16.0. A *P*-value<0.05 was considered statistically significant. 

## Results

***Light microscopic and TEM observations of fetal liver***

Examination of sections obtained from the livers of the control group revealed normal histological structure.

**Figure 1:**
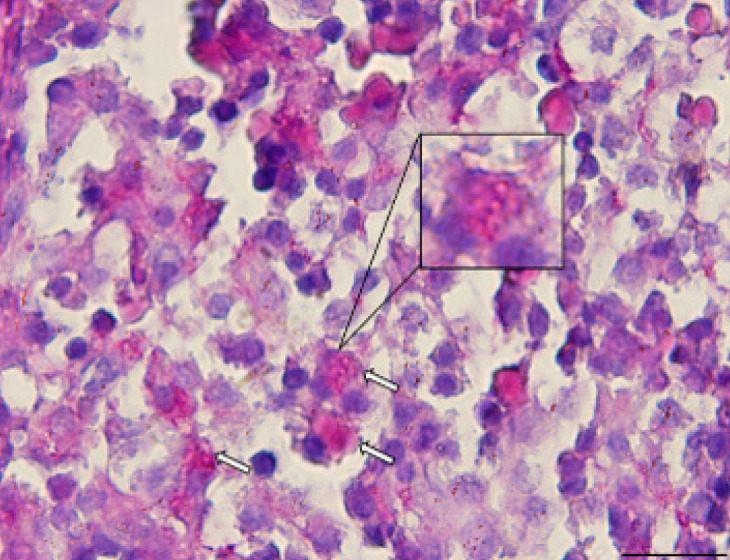
Photomicrograph of the rat fetal liver section in GDM group (PAS staining, ×100, scale bar = 20 µm)

PAS staining was positive, revealing scattered eosinophilic inclusions in some hepatocytes in the GDM group (Figure 1, white arrows), while it was negative in the control group.

**Figure 2:**
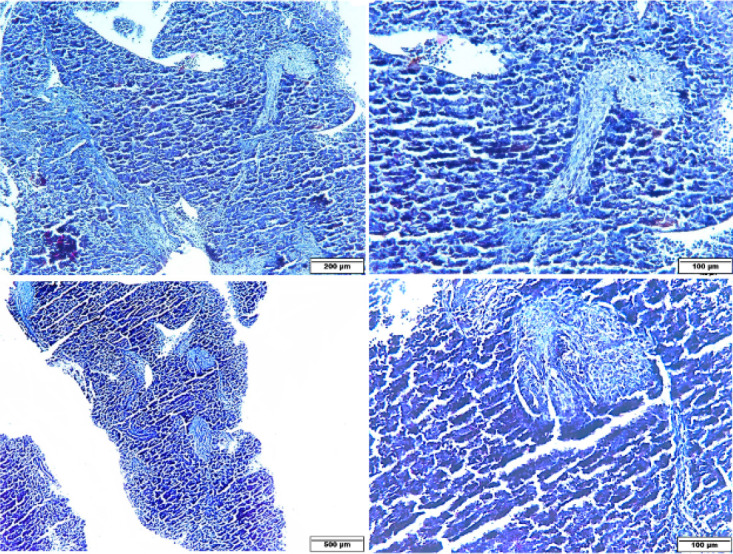
Photomicrographs of rat fetal liver sections in GDM group (Masson trichrome staining)

Masson trichrome staining was also positive and showed some fibrous tissue as fine fibers in the portal spaces that extended to the central vein (porto-central fibrosis) in the GDM group (Figure 2), while it was negative in the control group. 

**Figure 3:**
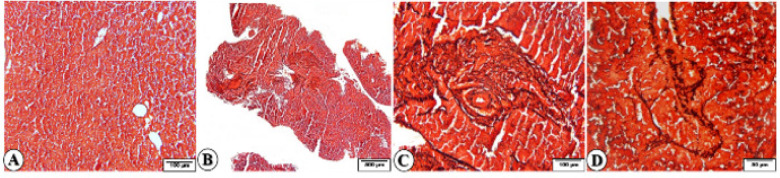
Photomicrographs of rat fetal liver sections

Reticulin staining was positive around the central vein and portal space vessels in the control group. In the GDM group, it was focally positive in the areas of fibrosis and the portal spaces (Figure 3).

**Figure 4:**
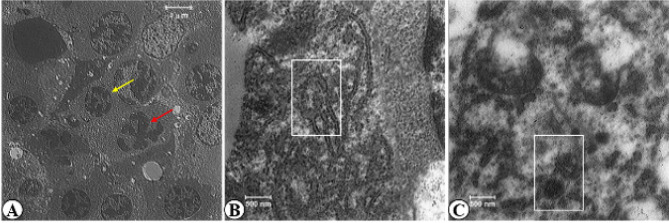
Transmission electron micrographs of rat fetal liver sections in GDM group

In the ultrastructural study, pyknotic nuclei followed by karyolysis, as well as degranulation and vesiculation of the rough endoplasmic reticulum (RER), along with distortion and degeneration of mitochondria were found in the GDM group compared to the control group (Figure 4).

***Morphometric results***

**Figure 5:**
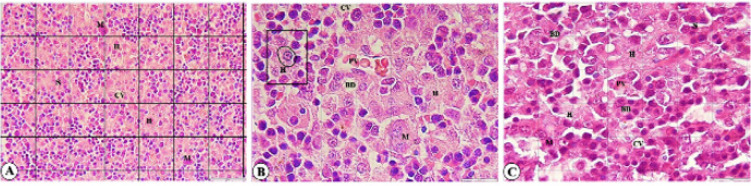
Photomicrographs of rat fetal liver sections

Morphometric parameters of fetal liver in GDM and control groups are given in Table 1 and Figure 5. The mean hepatocyte area and diameter were lower in the GDM group in comparison with the control group. These differences were statistically significant (mean difference area 14.03 ± 5.65 µm^2^ and diameter 1.02 ± 0.39 µm; *P*<0.05). However, there was no significant difference between groups in the nuclear area and diameter (mean difference area 0.78 ± 1.17 µm^2^ and diameter 0.09 ± 0.15 µm; *P*>0.05). A significant difference in the mean density of megakaryocytes between the groups was observed (mean difference between groups = -2.60 ± 0.54 No/100000 µm^2^; *P*<0.001). Furthermore, the mean diameter of the central vein in the GDM group was higher than that in the control group. This difference was statistically significant (mean difference between groups = -2.33 ± 0.67 µm; *P*= 0.001). No significant difference was found in bile duct diameter between the groups (mean difference between groups = -0.29 ± 0.33 µm; *P*=0.382). 

Furthermore, the correlation of hepatocyte area with the nucleus area and diameter in GDM and control groups is shown in Table 2. The parameters did not correlate in the control group, as shown by Pearson’s correlation coefficient test, while there was a strong positive correlation between hepatocyte area and nucleus area (r = 0.61, *P*=0.002) in the GDM group. 

## Discussion

In the present study, numerous histological, ultrastructural, and morphometric alterations were detected in the fetal liver following uncontrolled mild GDM.

These alterations included pyknotic nuclei, karyolysis, degranulation and vesiculation of the RER, and degeneration of mitochondria in the GDM group. Moreover, some fibrous tissue, as fine fibers in the portal spaces that extended to the central vein, was observed in the GDM group. 

Similar to our results, in a study by El-Sayyad *et al*., in which experimental DM was induced by two successive intraperitoneal injections of STZ (60 mg/kg b.w) on days 5 and 6 of gestation and pregnant rats with blood glucose levels > 350 mg/dl were included in the diabetic group, abnormal mitochondria and RER were detected in the liver of 19-day-old fetuses of diabetic dams. Also, moderate fibrotic change was found in the liver of diabetic dams (17).

In addition, increased fibrous tissue was observed after six weeks in the liver of diabetic male rats (60 mg/kg b.w., intraperitoneal injection of STZ) compared to control in the study of Alshathly *et al*. (18).

Despite the morphometric studies of the liver of adult diabetic rats, the quantitative aspects of fetal liver structure have been less investigated in this regard. In our study, the mean hepatocyte area and diameter were significantly lower in the GDM group in comparison with the control group. Moreover, the mean diameter of the central vein was significantly higher in the GDM group than in the control group.

Previous experimental studies have revealed morphometric changes in the liver of diabetic rats (19-22). The results of the study by Salahshoor *et al*. (19) demonstrated a significant increase in the size of hepatocytes and central vein in diabetic male Wistar rats (60 mg/kg b.w., intraperitoneal injection of STZ) compared to the normal control group.

In another study, the mean area of hepatocytes, nuclei, and nucleolus reduced in the periportal zone and increased in the perivenous zone in the diabetic male Wistar rats (STZ-induced DM, 80 mg/kg b.w., intraperitoneal injection). The increase of hepatocyte area in the perivenous zone and the decrease of nucleus area in the periportal zone were significant in the diabetic group compared to the control group (20).

In another study (21) the morphometric parameters of the liver (Hepatocyte diameter and Central hepatic vein) in diabetic male Wistar rats (STZ-induced DM, 60 mg/kg b.w., single intraperitoneal injection) were increased.

In addition, DM (60 mg/kg b.w., intraperitoneal injection of STZ) caused an increase in the size of hepatocytes and their nuclei with a decrease in the nucleus-to-plasma ratio in rat liver in another study (22).These differences may be due to the type of DM (mild GDM) and the survey of the liver in the fetal stage in our study.

Furthermore, in our study, the density of megakaryocytes was significantly higher in the GDM group than in the control group. Researchers reported abundant lymphocytes and megakaryocytosis in the liver of fetuses of diabetic rats (STZ-induced DM before pregnancy, 35 mg/kg b.w., intraperitoneal injection) on gestational day 20 (23).

Liver injury in GDM animal models can occur due to hyperglycemic conditions, which cause hyperinsulinemia and insulin resistance. Hyperglycemic conditions cause inflammatory conditions and oxidative stress and thus worsen the liver injury process by triggering NF-B activation, which stimulates the pro-apoptotic genes activity in liver cells and enhances the production of reactive oxygen species (24, 25).

**Table 1:** Morphometric evaluation of rat fetal liver in GDM and control groups

Parameters	Control (Mean ± SD*)	GDM (Mean ± SD*)	*P*-value
Hepatocyte area (µm^2^)	123.01±24.62	108.97±18.36	0.016^**^
Hepatocyte diameter (µm)	13.91±1.64	12.89±1.15	0.012^**^
Hepatocyte nuclei area (µm^2^)	31.85±5.12	31.06±5.67	0.504
Hepatocyte nuclei diameter (µm)	6.74±0.61	6.64±0.80	0.515
Central vein diameter (µm)	13.05±2.05	15.38±2.77	0.001^**^
Bile duct diameter (µm)	7.40±1.55	7.69±0.69	0.382
Density of megakaryocytes (No/100000 µm^2^)	2.06±1.33	4.67±2.47	>0.001^**^

*SD. Standard deviation; **. Significant level in *P*<0.05.

Hepatocyte diameter (long axis); Nucleus, central vein, and bile duct diameter (X, Y axes); Density of megakaryocytes: number of megakaryocytes in 100000 μm^2^

GDM: Gestational diabetes mellitus

**Table 2:** Correlation between the morphometric parameters of rat fetal liver in GDM and control groups

Group			Hepatocyte area
Control	Hepatocyte nuclei area	Pearson correlation	0.25
		Sig. (2-tailed)	0.158
	Hepatocyte nuclei diameter	Pearson correlation	0.03
		Sig. (2-tailed)	0.839
GDM	Hepatocyte nuclei area	Pearson correlation	0.61^**^
		Sig. (2-tailed)	0.002
	Hepatocyte nuclei diameter	Pearson correlation	0.50^*^
		Sig. (2-tailed)	0.013

**. Correlation is significant at the 0.01 level (2-tailed)

*. Correlation is significant at the 0.05 level (2-tailed)

GDM: Gestational diabetes mellitus

GDM: Gestational diabetes mellitus

GDM: Gestational diabetes mellitus

(A) Control group, (B–D) GDM group (Reticulin staining)

GDM: Gestational diabetes mellitus

(A) Pyknotic nuclei (yellow arrow) and karyolysis (red arrow), (B) degranulation and vesiculation of the RER, (C) distortion and degeneration of mitochondria.

GDM: Gestational diabetes mellitus

(A) Control group (H&E, ×40, scale bar = 50 µm), (B) Control group (H&E, ×100, scale bar = 20 µm), (C) GDM group (H&E, ×100, scale bar = 20 µm). Morphometric parameters: area and diameter of hepatocyte and nucleus, diameter of central vein and bile duct, and the density of megakaryocytes. Hepatocyte (H), central vein (CV), bile duct (BD), megakaryocyte (M), blood sinusoid (S), and portal vein (PV).

***Limitation***

There was no possibility of using ultrasound to determine the time of gestation in rats.

## Conclusion

This study highlighted the histological, ultrastructural, and morphometric alterations in the fetal liver due to uncontrolled mild GDM.
